# FoxP1 Stimulates Angiogenesis by Repressing the Inhibitory Guidance Protein Semaphorin 5B in Endothelial Cells

**DOI:** 10.1371/journal.pone.0070873

**Published:** 2013-09-02

**Authors:** Sebastian Grundmann, Christian Lindmayer, Felix P. Hans, Imo Hoefer, Thomas Helbing, Gerard Pasterkamp, Christoph Bode, Dominique de Kleijn, Martin Moser

**Affiliations:** 1 Department of Cardiology and Angiology I, University Heart Centre Freiburg – Bad Krozingen, Freiburg, Germany; 2 Experimental Cardiology Laboratory, University Medical Center, Utrecht, The Netherlands; 3 Interuniversity Cardiology Institute of the Netherlands, Utrecht, The Netherlands; University of Illinois College of Medicine, United States of America

## Abstract

Forkhead box (Fox) transcription factors are important regulators of cardiovascular development and several Fox-proteins have recently been shown to modulate embryonic and post-natal angiogenesis. However, the role of the FoxP subfamily, which is highly expressed in cardiovascular tissue, has not been investigated so far. Here, we show that the transcription factor FoxP1 is the highest expressed FoxP-protein in endothelial cells and that it is upregulated at the site of neovascularization during hindlimb ischemia in mice. Silencing of FoxP1 results in a strong inhibition of proliferation, tube formation and migration of cultured endothelial cells. Accordingly, knockdown of FoxP1 in zebrafish was followed by a disruption of intersomitic vascular formation. Using gene expression profiling, we show that FoxP1 induces a specific change of the endothelial transcriptome and functions as a suppressor of semaphorin 5B, which has previously been described as a neuronal inhibitory factor. Our findings now demonstrate that semaphorin 5B also acts as a FoxP1- dependent suppressor of endothelial cell proliferation, migration and sprouting, mediating the effects of FoxP1. In summary, our data indicate that the transcription factor FoxP1 is essential for the angiogenic function of endothelial cells and functions as a suppressor of the inhibitory guidance cue semaphorin 5B, suggesting an important function of FoxP1 in the regulation of neovascularization.

## Introduction

The development and growth of capillary and arterial blood vessels is a basic process in embryonic development as well as during physiological and pathophysiological processes in the adult organism, such as the neoplastic tumor growth, inflammation, wound healing and the adaptive response to tissue ischemia due to atherosclerotic vascular disease. The regulation of these adaptive processes is a complex event, orchestrated by transcriptional factors that regulate the expression of both pro- and anti-angiogenic genes [1]. Recently, the Forkhead box (Fox) class of transcription factors was implicated in this process [2]. The Fox proteins constitute a large group of transcriptional regulators that are characterized by an evolutionary conserved “winged helix” DNA-binding domain [3]. The more than 100 different Fox-proteins are divided into subgroups based on their sequence homology and are of critical importance for different aspects of embryonic development and various postnatal cellular processes. So far, three families of Fox-proteins have been studied in the context of blood vessel development and neovascularization: the FoxO-family, FoxH1 and FoxC.

First, following the discovery of embryonic lethality of FoxO1-deficient mice by Furuyama et al. [4], Potente and co-workers demonstrated that FoxO1 and FoxO3a are the strongest expressed FoxO-isoforms in endothelial cells and that both exhibit an inhibitory function on angiogenesis [2]. For FoxH1, Choi et al. recently showed that the VEGF-receptor-2 (flk1/kdr) has a conserved FoxH1 binding-site and that overexpression of this transcription factor in zebrafish embryos results in a disruption of vascular pattern formation [5]. Seo et al. described the role of FoxC1 in inhibiting corneal angiogenesis by regulating VEGF signalling [6]. Despite a strong expression in cardiovascular tissue and an important function in the development and adaptive remodelling of the endocardium [7], the FoxP subgroup has not been studied in the context of blood vessel growth before. This small subgroup of the Fox family contains four members (FoxP1-4) that function predominantly as transcriptional repressors with very different functions in a diverse range of physiological and pathophysiological processes, ranging from speech development (FoxP2) [8] to immunomodulation in regulatory T-cells (FoxP3) [9] and cardiogenesis (FoxP1, FoxP4) [10]. Besides the winged-helix forkhead domain, the FoxP subgroup is characterized by a zinc-finger and a leucin-zipper motif in the N-terminus, allowing the formation of heterodimers with other forkhead proteins [3]. In the present study, we now show for the first time that FoxP1 is the highest expressed FoxP-family member in endothelial cells and study its function in endothelial cell biology and angiogenesis. This transcription factor was first identified by Li et al. [11] and shows a strong expression in a wide selection of tissues, with the highest expression in lymphoid and gastrointestinal samples. It is expressed in at least four splice isoforms. Although sequence differences suggest potential functional differences between the isoforms, little is known about their distinct expression patterns and regulatory properties, which can differ between individual cell types. E.g. Foxp1A and Foxp1D repress the IL-2 promoter at similar levels in Jurkat cells, whereas Foxp1D is significantly stronger than Foxp1A in 293T cells [3]. In malignant neoplastic tissue, FoxP1 shows a heterogeneous expression pattern [12]. A special importance of FoxP1 in B-cell lymphopoiesis was recently identified by Hu et al. [13] and overexpression of this transcription factor in a subset of diffuse large B cell lymphoma indicates a poor prognosis for this disease.

FoxP1 was first mentioned In the cardiovascular field by Wang et al, describing the phenotype of genetic FoxP1 deficiency in mouse embryonic stem cells [7]: inactivation of Foxp1 resulted in severe cardiac defects, including defective ventricular and outflow tract septation and valve formation. While indirect observations, such as perivascular hemorrhage, indicated a potential involvement of FoxP1 in vascular development, the dominant cardiac phenotype did not allow a specific investigation of vascular growth in these embryos.

Our data now indicate a pro-angiogenic role of FoxP1 on endothelial cell proliferation, migration and angiogenic tube formation as well as vascular smooth muscle cell proliferation. In addition, we describe the modulation of the endothelial cell transcriptome by FoxP1, including the suppression of the inhibitory guidance molecule semaphorin 5B.

## Methods

*An additional methods section is available in the online in the file Methods S1.*

### Cell culture

The investigation conforms to the principles outlined in the Declaration of Helsinki for the use of human tissues. Human umbilical vein endothelial cells (HUVECs) were isolated and cultivated as previously described [14] in endothelial basal medium supplemented with FCS-kit single quots (Provitro, Berlin, Germany) plus 10% FBS and used until passage five. Human aortic vascular smooth muscle cells (No. CRL-1999) were obtained from the ATCC and cultured under the recommended conditions.

### Plasmid transfection and RNA interference

A FoxP1 constructs expressing the transcript variant 1 of human FoxP1 (NM_032682.4) under the control of the constitutively active CMV-Promotor as well as a myc-DDK-tagged ORF clone of human semaphorin 5B (NM_001031702.2) and the appropriate control constructs (pCMV6-XL5) were obtained from Origene Technologies (Rockville, MD). For transient transfections, DNA plasmids were introduced into HUVECs using PromoFectin-HUVEC transfection reagent according to the manufacturer's instructions (Promocell). FoxP1 siRNA was obtained from Invitrogen (For siRNA sequences see Methods S1). Scrambled negative control-Alexa Fluor 488 nm was purchased from Qiagen. For siRNA transfection, Lipofectamine RNAiMAX was used according to the manufacturer's protocol (Invitrogen).

### RNA isolation and real-time PCR analysis

For quantitative analysis of mRNA-levels, RNA was isolated according to standard protocol (RNAeasy kit, Qiagen), 1 µg total RNA was transcribed to cDNA (iScript, Biorad) and real-time PCR was performed on a MyIQ (Biorad) as previously described [15]. The expression of beta-actin, hRPII or the ribosomal RNA 18S served as a loading control. All measurements were performed in duplicates.

### Proliferation assay

Proliferation was assessed using a colorimetric BrdU-incorporation ELISA (Roche). 24 h after transfection, cells were cultured in fresh BrdU-containing medium for another 24 h. The colorimetric ELISA for BrdU quantification was performed following the manufacturers instruction.

### Tube formation assay

24 h following transfection for silencing or overexpressing FoxP1, these assays were performed as previously described [14]. All experiments were performed at least three times.

### Planar migration assay

The wound healing planar migration assay was performed at 12 h or 16 h after transfection with FoxP1 siRNA or FoxP1 expressing plasmid as previously described [16].

### Microarray analysis

Total RNA was isolated from HUVECs 48 h after transfection with a mix of two different FoxP1 siRNAs or a control siRNA as described above. Gene expression profiles of three independent samples per condition were assessed with the Agilent human 4×44K microarray system. A detailed methodology and complete raw data level of the microarray results were deposited at the NCBI gene expression and hybridization array data repository (GEO, http://www.ncbi.nlm.nih.gov/geo/) and can be accessed under the Accession number GSE 32968.

### Western blotting

For western blot analysis of FoxP1 or semaphorin 5B expression, cultured cells were lysed in RIPA buffer on ice and protein concentration was quantified using the Bradford method. Equal amounts of protein were loaded, subjected to SDS-PAGE and blotted on nitrocellulose membranes. Blots were incubated over night with antibodies directed against human FoxP1 (mouse monoclonal, clone JC12, Abcam, Cambridge, UK) or against semaphorin 5B (goat polyclonal, sc-67959, Santa Cruz biotech., Santa Cruz, CA, USA). Beta-tubulin and GAPDH were used as loading controls.

### Murine hindlimb ischemia model

The study conforms to the *Guide for the Care and Use of Laboratory Animals* published by the U.S. National Institutes of Health and was performed after securing approval of the local animal care and use committee (Regierungspräsidium Freiburg). Unilateral femoral artery occlusion was performed in C57/Bl6J mice (Charles River Lab., Sulzfeld, Germany) under anaesthesia with ketamin and xylazin by double ligation of the superficial femoral artery proximal to the deep femoral artery and the distal femoral artery just above the knee joint as previously described [17], [18]. Tissue of the gastrocnemius and soleus muscles was carefully dissected from the lower leg and snap-frozen in liquid nitrogen until further analysis.

### Statistical analysis

Data are expressed as mean and SEM. Treatment groups were compared by Student's t-test using Prism 4 for Windows (GraphPad Software Inc). ANOVA with Bonferroni correction was used for multiple comparisons of more than two groups, if the P value for the overall ANOVA comparison was statistically significant. P-values <0.05 were considered to be statistically significant.

## Results

### FoxP1 is the predominant FoxP transcription factor in endothelial cells

**Figure 1:**
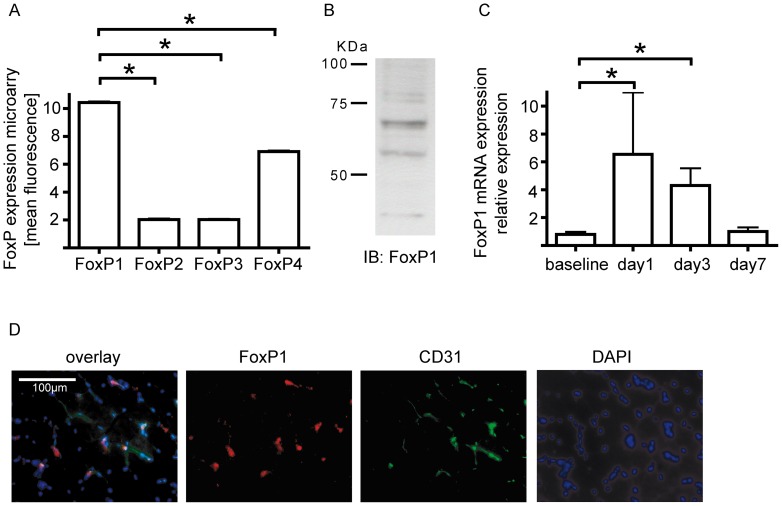
Foxp1 is highly expressed in endothelial cells and upregulated during blood vessel growth in vivo. Microarray analysis of Forkhead box P transcription factor expression in human umbilical vein endothelial cells showed the highest expression for the FoxP1 subtype (A), with the highest expression of the 68kDa splice variant on protein level (B). (* = p<0.05) In vivo, FoxP1 expression is upregulated at the site of neovascularization upon induction of hindlimb ischemia in mice (C). Immunofluorescent double staining of FoxP1 (red) with the endothelial marker CD31 (green) and a nuclear staining (DAPI, blue) localized FoxP1 expression to endothelial cells of the capillary vasculature in the gastrocnemius muscle of the mouse hindlimb (D).

To assess the potential involvement of FoxP transcription factors in endothelial biology, we first analyzed the baseline expression of FoxP1-4 in human umbilical vein endothelial cells. Microarray analysis of unstimulated HUVECs cultured under standard conditions showed that FoxP1 is the predominant FoxP isoform expressed in HUVECs, whereas FoxP4 shows the second highest expression (Figure 1A). Western blotting revealed the presence of two distinct variants of FoxP1 in endothelial cells, with the isoform displaying at approx. 68 kDa showing the highest expression (Figure 1B).

### FoxP1 is upregulated after induction of hindlimb ischemia in mice

Since FoxP1 is highly expressed in endothelial cells, we next asked if this transcription factor is differentially expressed during vascular proliferation in vivo. We therefore performed a unilateral occlusion of the proximal femoral artery in mice, which results in the induction of downstream tissue ischemia in the lower leg. Quantification by real-time PCR analysis revealed a significant upregulation of FoxP1 in the ischemic hindlimb compared to the sham-operated contralateral control hindlimb (Figure 1C), demonstrating a modest physiological upregulation of this transcriptional regulator after induction of neovascularization in vivo. Within the ischemic tissue, immunohistochemistry localized FoxP1 expression predominantly to endothelial cells (Figure 1D).

### FoxP1 stimulates endothelial cell proliferation, migration and tube formation

**Figure 2:**
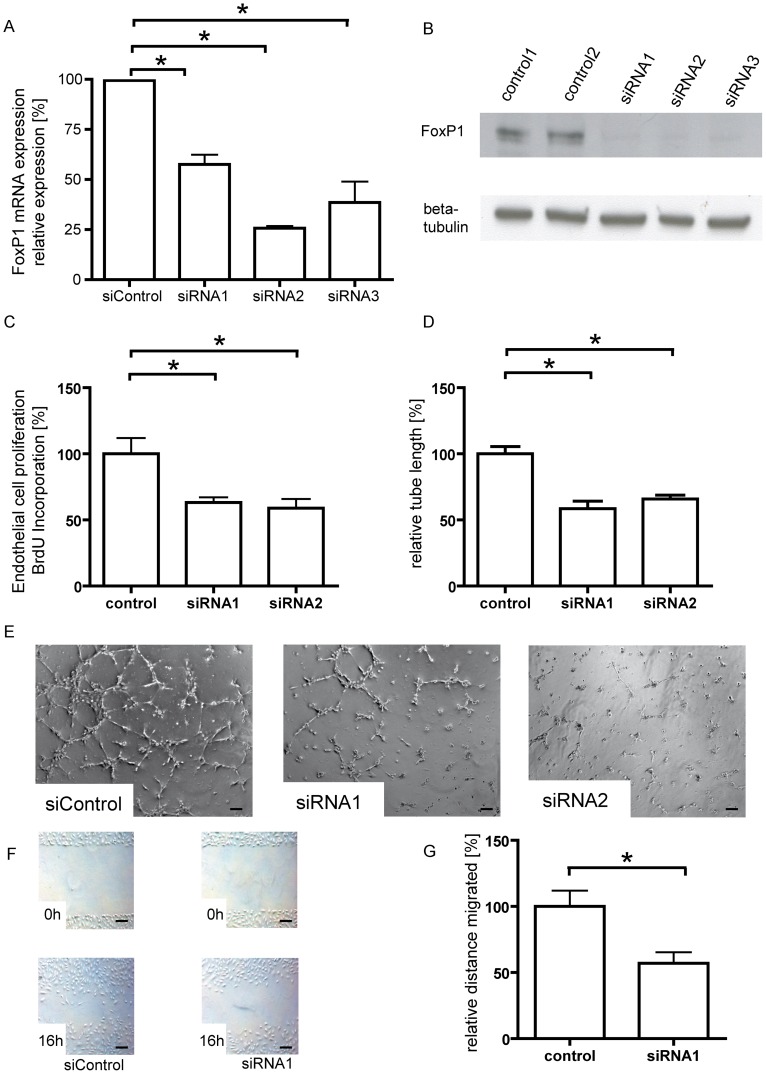
FoxP1 knockdown attenuates endothelial cell proliferation, tube formation and migration. Human umbilical vein endothelial cells were transfected with three different siRNA oligonucleotides against FoxP1, which resulted in a significant reduction of FoxP1 expression on both mRNA (A) as well as protein level (B) compared to transfection with a control siRNA. (C) Reduction of FoxP1 levels by two different siRNAs resulted in a significant attenuation of endothelial cell proliferation. (D&E) For quantification of endothelial tube formation, transfected cells were cultured on matrigel and cumulative sprout length of capillary-like structures was measured after 3 hours. FoxP1 knockdown attenuated angiogenic tube formation, with a shorter total tube length compared to transfection with a control siRNA. (F+G) FoxP1 knockdown resulted in a significant inhibition of endothelial cell migration in the planar wound healing assay. (* = p<0.05, Scale bars: 100 µm).

As FoxP1 was highly expressed in endothelial cells and upregulated following induction of blood vessel growth in vivo, our next aim was to investigate the direct effect of FoxP1 on angiogenic endothelial cell function by loss-of-function and gain-of function experiments. Therefore we transfected HUVECs with siRNA against FoxP1 and achieved a sustained knockdown of FoxP1 mRNA and protein levels for up to 48 h by two different siRNAs (Figure 2 A+B). This reduction of FoxP1-levels resulted in a strong attenuation of endothelial cell proliferation as assessed by BrdU-incorporation (Figure 2C) and a lower total tube length in the two-dimensional matrigel assay (Figure 2 D+E). FoxP1 knockdown also resulted in the significant attenuation of endothelial cell migration in the planar wound healing assay (Figure 2 F–G). These three cell culture assays represent the three essential components of angiogenesis (proliferation, migration, tube formation), demonstrating that loss of FoxP1 results in reduced neovascularization.

**Figure 3:**
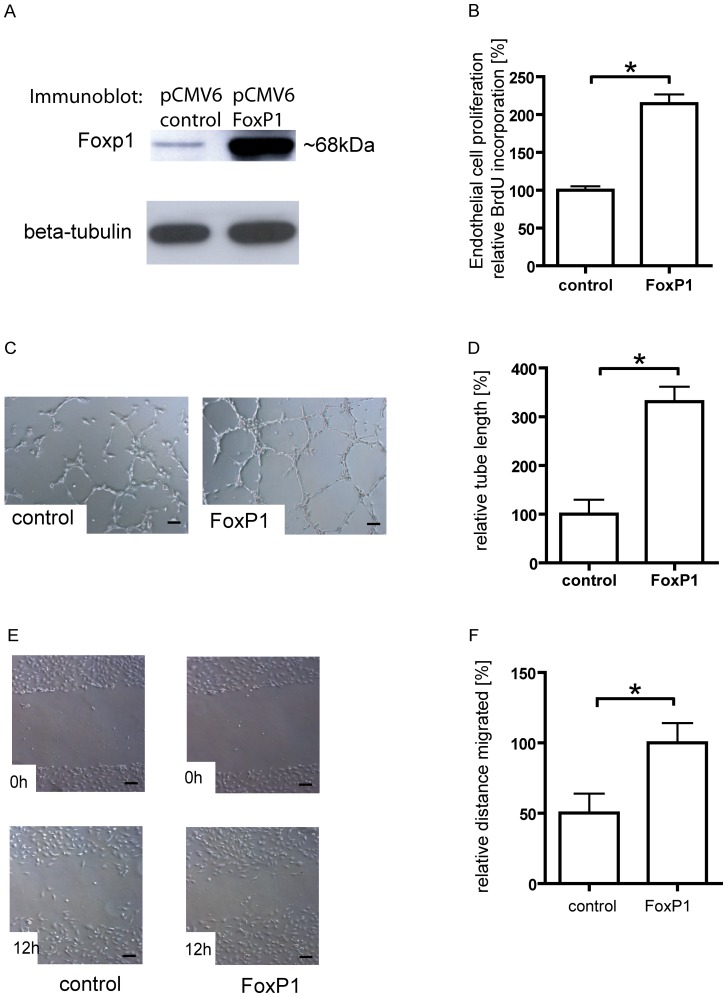
FoxP1 overexpression stimulates EC proliferation, tube formation and migration. (A) Transfection with a FoxP1-coding plasmid resulted in the strong overexpression of FoxP1. (B) FoxP1 overexpression stimulated endothelial cell proliferation and tube formation, with an increased cumulative sprout length of capillary-like structures (C–D). Also, in good correspondence to the observed attenuation of endothelial cell migration after FoxP1 knockdown, overexpression of FoxP1 resulted in a modest increase in migration distance in the planar wound healing assay (E–F). (* = p<0.05, Scale bars: 100 µm).

Based on these findings, we hypothesized that overexpression of FoxP1 should have the opposite effect. We therefore overexpressed the 68kD isoform as the predominant splice variant of FoxP1 in HUVECs, by transfecting a construct expressing this isoform under control of the constitutively active CMV-promoter (Figure 3A). Cellular viability after transfection did not significantly differ between cells transfected with an empty control vector and the FoxP1-overexpression construct. Corresponding to the observed inhibition of angiogenic function after FoxP1 knockdown, this gain-of-function approach now resulted in the stimulation of endothelial cell proliferation (Figure 3B) and tube formation in the matrigrel assay with a longer total tube length (Figure 3C–D). Endothelial cell migration in a planar wound healing assay was also stimulated (Figure 3E–F).

Taken together, FoxP1 is a positive regulator of pro-angiogenic endothelial cell function *in vitro*.

### FoxP1 knockdown attenuates vascular smooth muscle cell proliferation

Since the proliferation of vascular smooth muscle cells is an essential component of the growth of arteriolar and arterial blood vessels, we analyzed the proliferation rate after siRNA-mediated FoxP1 knockdown in this second vascular cell type. FoxP1 knockdown significantly attenuated the proliferation of vascular smooth muscle cells (Figure S1), measured by the amount of BrdU-incorporation. Thus, these findings indicate that FoxP1 stimulates not only endothelial but also smooth muscle cell proliferation.

### FoxP1 is essential for vascular pattern formation in zebrafish

Our next step was to investigate the potential functional importance of FoxP1 for the regulation of blood vessel growth *in vivo*. FoxP1-deficient mice have been reported to die early during embryonic development due to cardiac malformations [7], prohibiting the selective investigation of vascular development in this model. As FoxP1 is evolutionary highly conserved among vertebrate species, we therefore investigated blood vessel formation in *Tg (vegfr4:gfp)^s843^* transgenic zebrafish (danio rerio). This transgenic strain expresses green fluorescent protein under control of the endothelial-specific promoter of the VEGF-receptor kdr, allowing for direct observation of blood vessel formation using fluorescence microscopy. Two morpholinos (MOs) were designed to block mRNA-splicing of both FoxP1 paralogues (FoxP1a and FoxP1b) that exist in the zebrafish and resulted in either the generation of a mis-spliced band (MO1) or the reduction of wildtype FoxP1 transcript to 20% of uninjected controls (MO2). Development of the GFP-labeled blood vessels was observed in the live fish for 5 days.

**Figure 4:**
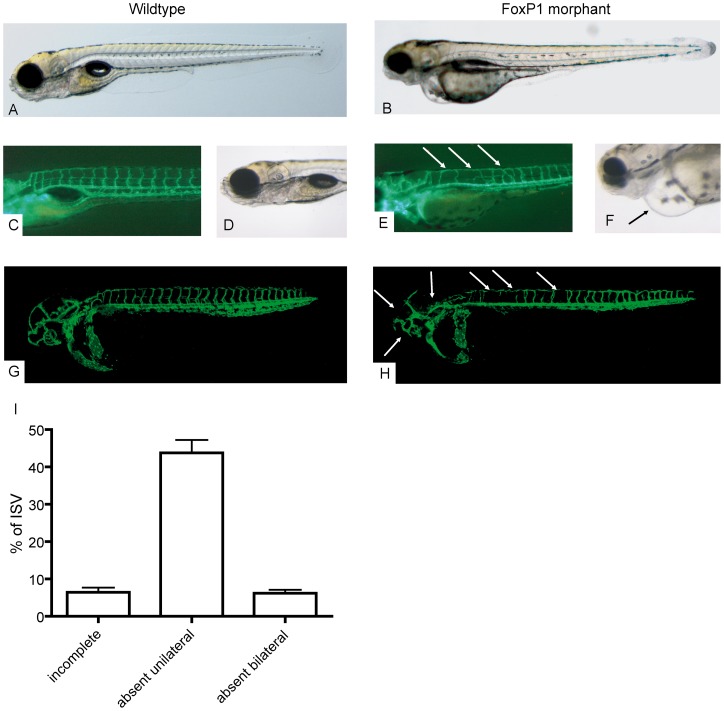
FoxP1 modulates blood vessel development in vivo. (A&B) No gross developmental defects were observed after FoxP1 silencing in *Tg (vegfr4:gfp)^s843^*fish, that allow fluorescent imaging of cardiovascular development. The combined silencing of the two zebrafish paralogues of FoxP1 FoxP1a and FoxP1b by injection of two morpholinos resulted in a specific and reproducible cardiovascular phenotype. FoxP1 knockdown impaired vascular (E) and cardiac (F+G) development. At day 5 post fertilization, most ISVs of the trunk remained missing or incomplete (E). Confocal imaging (G&H) revealed that most ISV that eventually sprouted from the dorsal aorta did not follow the normal intersomitic pathways, but penetrated intersomitic boundaries (depicted by arrows, Quantification: I). Cerebrovascular development was also impaired (H).

While the isolated knockdown of a single paralogue induced inconsistent morphological changes, the combined silencing of FoxP1a and FoxP1b by simultaneous injection of two MOs resulted in a specific and reproducible cardiovascular phenotype. In good correspondence with the previously published findings in mice and supporting the specificity of our approach, FoxP1a/b knockdown impaired cardiac development and resulted in a string-like heart with reduced cardiac fraction and pericardial effusion (Figure 4). In addition, we observed a consistent defect in vascular network formation: at day one post fertilization (dpf), silencing of FoxP1a and FoxP1b resulted in missing or misdirected intersomitic vessels (ISV) predominantly in the trunk. With the continuation of development, most ISVs of the trunk remained missing and most ISV that eventually sprouted from the dorsal aorta did not follow the normal intersomitic pathways, but penetrated intersomitic boundaries and even crossed the adjacent ISV before connecting to the dorsal longitudinal anastomotic vessel. At three dpf, 90% of the morphants showed this aberrant ISV pattern that persisted until day 5, the last time point observed.

Thus, while the generation of endothelial cells was not significantly impaired in the FoxP1-morphants, loss of FoxP1 results in defective vascular pattern formation and sprouting in zebrafish.

### Transcriptome analysis after FoxP1 knockdown and regulation of Semaphorin 5B by FoxP1 in endothelial cells

Following the discovery that FoxP1 stimulates proliferation, network formation and migration of vascular cells, we next asked how these effects might be mediated. Knowing that FoxP1 modulates cellular functions by transcriptional regulation, we analyzed the transcriptome of endothelial cells at 48 h after silencing of FoxP1 by siRNA-transfection using cDNA-microarray technology.

**Figure 5:**
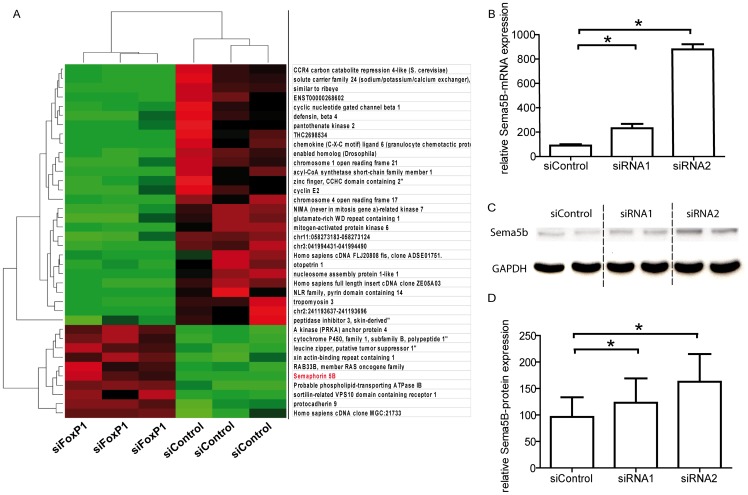
Semaphorin 5B expression is repressed by FoxP1 in endothelial cells. Microarray-based transcriptome analysis of endothelial cells 48 h after FoxP1 knockdown revealed a total of 10 upregulated and 28 downregulated genes. (A) The heat map shows a two-dimensional clustering of these genes, with expression intensities represented by red and green, for high and low intensities, respectively. Black indicates medium intensities. The inhibitory guidance protein semaphorin 5B was selected for further analysis because of the common link of semaphorins and FoxP1 to neuronal cell guidance and pattern formation. The suppression of Semaphorin 5B by FoxP1 could be validated on mRNA (B) and protein level (C: Western blot; D: densitometric quantification), as FoxP1 knockdown by different siRNAs resulted in an increase in semaphorin 5B expression. (* = p<0.05).

A global statistical analysis for differentially expressed genes after FoxP1 knockdown was performed with student's t-test, a significance level of 5% and a fold-change cut-off of 2. This analysis revealed a total of 10 upregulated and 28 downregulated genes at 48 h after silencing of FoxP1 (Figure 5A).

Among this short list of 10 significantly upregulated genes was the axonal guidance protein semaphorin 5B (Sema5B). Confirming our primary microarray results by PCR and Western blotting, we detected a low baseline expression of semaphorin 5B in endothelial cells, but a significant increase in expression on both mRNA and protein level after FoxP1 knockdown (Figure 5B–D). Therefore, we chose Sema5B for further analysis as a potential mediator of the observed pro-angiogenic effects of FoxP1.

### Semaphorin5B is an inhibitory factor for endothelial proliferation, migration and tube formation

**Figure 6:**
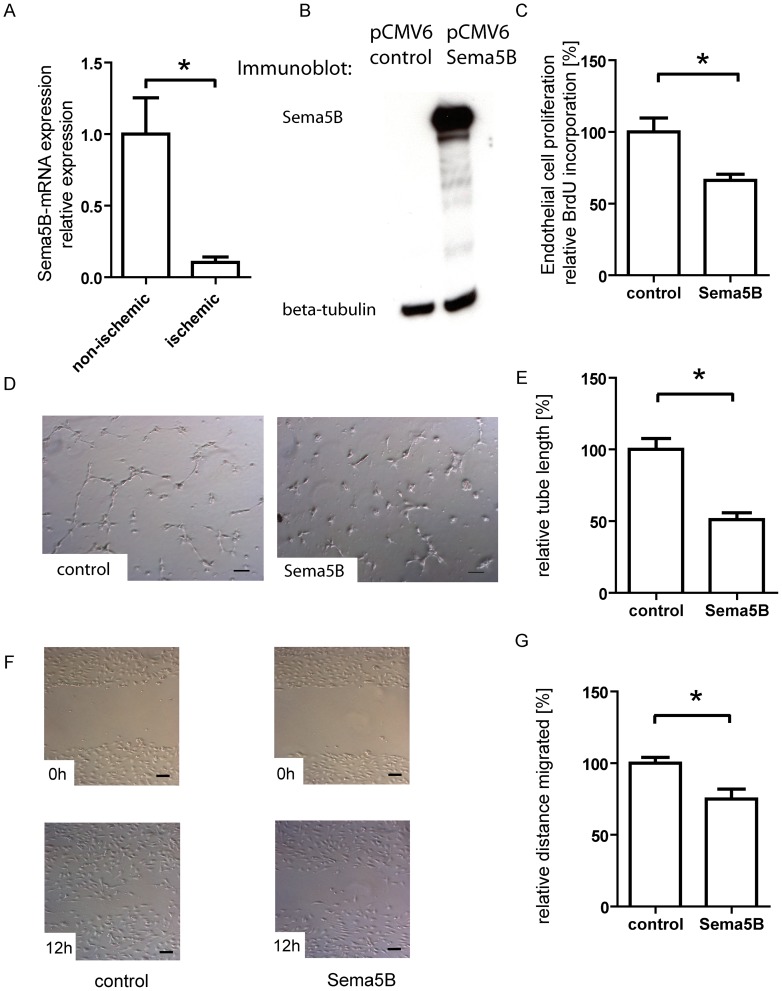
Semaphorin 5B is downregulated after induction of ischemia in vivo and inhibits endothelial cell proliferation, migration and tube formation. (A) In vivo, semaphoring is downregulated after induction of ischemia by femoral artery ligation in mice. (B) Transfection with a semaphorin 5B coding plasmid resulted in a strong overexpression of this cytokine in endothelial cells. In functional cell culture assays, semaphorin 5B overexpression mimicked the effects of FoxP1 knockdown and impaired angiogenic functions of endothelial cells. Specifically, semaphorin 5B overexpression attenuated endothelial cell proliferation as measured by BrdU-incorporation (C) and endothelial tube formation on the planar matrigel assay (D), resulting in a shorter total tube length (E). Migration in the wound healing assay was also impaired (F&G). (* = p<0.05, Scale bars: 100 µm).

We detected a significant downregulation of semaphorin 5B in mouse hindlimb tissue after induction of ischemia, corresponding to the observed upregulation of FoxP1 and therefore in agreement with our hypothesis that FoxP1 acts as Semaphorin 5B repressor (Figure 6A). As semaphorin 5B is well described as an inhibitory guidance cue for neuronal axons [19] and after we had confirmed its regulation by FoxP1, we investigated if this protein has an inhibitory effect on angiogenic functions of endothelial cells as well. Therefore, we overexpressed semaphorin 5B in endothelial cells, resulting in a strong increase in semaphorin 5B levels (Figure 6B). Semaphorin 5B overexpression strongly attenuated the proliferation (Figure 6C), tube formation (Figure 6D–E) and planar migration (F–G) of endothelial cells. This is in agreement with the effects observed after FoxP1 knockdown, where we could show semaphorin 5B levels to be increased.

## Discussion

We describe the increased expression of the Forkhead box protein P1 during neovascularization and investigated for the first time the role of this transcription factor in endothelial cell biology. Our data indicates a role of FoxP1 as a stimulator of angiogenesis by promoting endothelial proliferation, migration and endothelial tube formation as well as its function as a suppressor of the inhibitory guidance cue semaphorin 5B.

With FoxP1, we now add the first member of the FoxP-subfamily to the forkhead transcription factor group of vascular regulators. While the functional role of FoxP1 has so far been investigated in circulating cell populations and in neurons, we now describe for the first time a pro-angiogenic role of this transcription factor in endothelial cells. Using both loss of function as well as gain of function approaches, we found that FoxP1 stimulates the proliferation, migration and tube formation of endothelial cells in several established cell culture angiogenesis assays, indicating a significant regulatory function of FoxP1 in endothelial and vascular smooth muscle cells.

Since FoxP1 deficiency results in early intra-uterine lethality in mice and the defective cardiac development in these embryos prohibits the characterization of the vascular phenotype, we used a kdr:GFP transgenic zebrafish line to investigate the importance of FoxP1 for vascular formation in vivo. In this organism, tissue oxygenation and vasculogenesis is independent of cardiac function during early embryonic development, enabling the study of vascular development under conditions of FoxP1 deficiency using morpholino-mediated knockdown.

Indeed, next to the previously described impaired cardiac development, we observed a consistent defect in vascular pattern formation, predominantly of the truncal intersomitic blood vessels.

Following these findings, our next step was to elucidate the mechanism by which this transcriptional regulator achieves these effects. Using microarray based analysis of endothelial cells after FoxP1 knockdown, we detected a specific modulation of the endothelial transcriptome (Figure 4). We selected genes for further analysis by a sequential approach: as FoxP1 is known to function predominantly as a transcriptional repressor [3], we focused primarily on genes upregulated after FoxP1 knockdown. This relatively short list of 10 genes, showed a very similar moderate degree of suppression, with a fold change between 2.0 and 2.25. Among this group was the inhibitory guidance protein semaphorin 5B, which we chose for further investigation because of the common link of semaphorins and FoxP1 to axonal guidance and neuronal pattern formation [19], [20], [21]. This process of axonal path finding shares many regulatory mechanisms with vascular proliferation and pattern formation [22], [23], and several mediators of vascular sprouting were subsequently discovered to exert a similar function in neuronal connectivity [24].

We now identified FoxP1 as an inhibitor of semaphorin 5B expression in endothelial cells and validated our primary microarray results on both mRNA and protein level, as semaphorin 5B levels increased rapidly after siRNA-mediated FoxP1 knockdown. While other studies on the regulatory functions of forkhead transcription factors frequently used overexpression of the transcription factor as the independent variable, we opted for a gene-silencing approach, as we already detected high endogenous FoxP1 levels under cell culture conditions in human umbilical vein endothelial cells.

Semaphorin 5B was first cloned by Adams and coworkers in 1996 [25] and in the human fetus, this protein shows an intermediate expression in heart, brain, lung, kidney, ovary, testis, spinal cord, fetal liver and the fetal brain [26]. The still growing family of semaphorins is characterized by the approximately 500 amino acid long sema domain, which is essential for their activity and receptor binding specificity [27]. Semaphorin 5B was recently described as a repulsive neural guidance cue, which is proteolytically processed from its transmembrane form [28]. While several semaphorin family members have already been shown to exert anti-angiogenic functions [29], [30], [31], we now provide evidence for an inhibitory effect of semaphorin 5B on endothelial cell proliferation, migration and tube formation. Besides the sema-domain, class 5 semaphorins are characterized by additional thrombospondin repeats, similar to the type 1 TSP repeats found in thrombospondin-1 and -2, which could potentially mediate this anti-angiogenic function. These domains contain specific peptide sequences able to bind the endothelial cell surface receptor CD36, thereby inhibiting angiogenesis *in vitro* and *in vivo* [32].

Our study describes a regulatory role of FoxP1 in adaptive neovascularization and demonstrates the modulation of anti-angiogenic gene expression as the potential mechanism of this FoxP1-function. However, the precise mechanism of FoxP1 regulatory function and the necessary co-factors in endothelial cells remain to be elucidated. Our results clearly demonstrate that knockdown of FoxP1 levels by siRNA-transfection in endothelial cells results in the upregulation of semaphorin 5B. Still, the semaphorin 5B promoter region region has not been characterized yet and a direct regulation by FoxP1 cannot be concluded from our data. If FoxP1 inhibits the anti-angiogenic function of this protein by direct binding to the semaphorin 5B promoter region or via an indirect regulatory element therefore remains to be elucidated. Although immunohistochemistry localized FoxP1 expression to endothelial cells in vivo, its expression is not cell type specific and regulatory functions in bone marrow derived cell populations are well described [33], [34]. As these circulating cells infiltrate ischemic tissue, a direct conclusion about the cell population responsible for the increase in FoxP1 expression after femoral artery ligation is not possible from our data.

Taken together, our findings suggest an important pro-angiogenic function of the Forkhead box protein P1 by repressing the inhibitory guidance cue semaphorin 5B in endothelial cells.

Further investigation of the common regulatory mechanisms of neuronal and vascular growth could yield more targets for the positive or negative modulation of blood vessel growth.

## Supporting Information

Figure S1 **Knockdown of FoxP1 by two different siRNAs attenuates vascular smooth muscle cell proliferation.**
(PDF)

Methods S1 **Additional description of methods and reagents.**
(PDF)
