# Supplementary figures and images for: FoxP1 Stimulates Angiogenesis by Repressing the Inhibitory Guidance Protein Semaphorin 5B in Endothelial Cells
Source: PLoS One. 2013 Sep 2;8(9):e70873. doi: 10.1371/journal.pone.0070873 (PMC3759435; doi:10.1371/journal.pone.0070873)

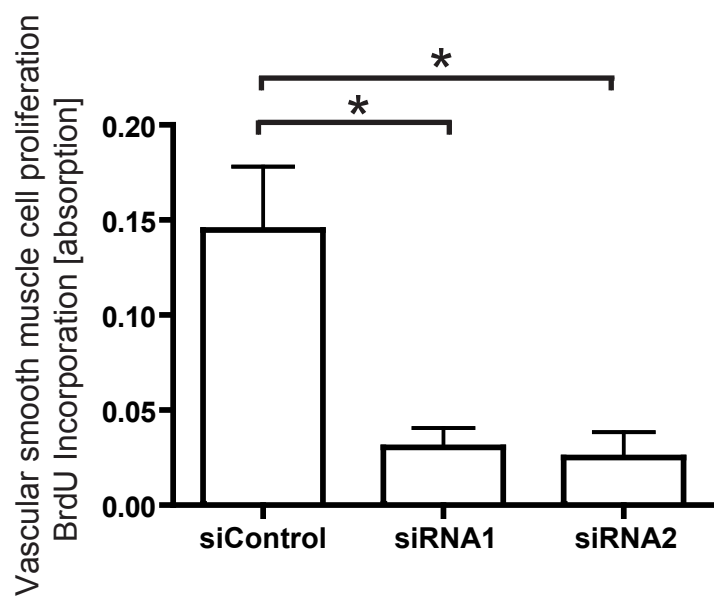

**Supplementary Figure 1:** *Foxp1* knockdown attenuates vascular smooth muscle cell proliferation

Supplement: Figure S1 — Knockdown of FoxP1 by two different siRNAs attenuates vascular smooth muscle cell proliferation. (PDF) [file pone.0070873.s001.pdf]
