# Supplementary material for: FoxP1 Stimulates Angiogenesis by Repressing the Inhibitory Guidance Protein Semaphorin 5B in Endothelial Cells
Source: PLoS One. 2013 Sep 2;8(9):e70873. doi: 10.1371/journal.pone.0070873 (PMC3759435; doi:10.1371/journal.pone.0070873)
Supplement: Methods S1 — Additional description of methods and reagents. (PDF) [file pone.0070873.s002.pdf]

Supplemental Methods

Histology

For immunohistology, 5µm frozen tissue sections were processed as previously described[5], incubated with the appropriate primary and secondary antibodies (see supplementary table 1) and embedded in moviol. DAPI dye was used for nuclear staining. Imaging was performed on a Zeiss Axioplan microscope and images where processed with Adobe Photoshop 7.0 for contrast enhancement.

Zebrafish Stocks, Morpholino Design, Phenotype Analysis and Detection of Splice Variants of Zebrafish FoxP1 mRNA with RT-PCR

The wild-type AB strain and *Tg(vegfr4:gfp)*^{s8436} (originally referred to as *Tg(flk1:EGFP)*^{s843}) transgenic zebrafish were used in this study. Two splice-blocking morpholinos for zebrafish FoxP1a (MA1 and MA2) and its predicted paralogue FoxP1b (MB1 and MB2) were synthesized by Gene-Tools (Philomathe, OR). Morpholinos were dissolved in nuclease-free water at 50mM and injected into 1-2-cell stage embryos. The injected amount was determined by measuring the size of the liquid sphere injected into oil. Injected embryos were cultured in fish water at 28.5°C. For detection of splice variants, total RNA of was isolated at 48hpf using Tripure and transcripted to cDNA as described below. Splice variants were visualized on agarose-gel and total Foxp1 mRNA was quantified by real-time RT-PCR as previously described [4]. The cardiovascular phenotype of the FoxP1 morphants was investigated by life microscopic imaging of the transgenic embryos and by confocal microscopy.

Reagents

PCR-primers

**gene**	**species**	**forward (5'-> 3')**	**reverse (5'-> 3')**
FoxP1	human	tcagtggtaacccctttta	gtacaggatgcacggctt
Sema5b	human	gctggcttctctcactggtc	agctggagaggtgggacac
ß-actin	human	gatcggcggctccatcct	gactcgtcatactcctgcttgc

primary antibodies

**against:**	**host**	**Cat #**	**company**
FoxP1	rabbit	ab16645	abcam
Sema5b	goat	sc-67959	Santa Cruz Biotech
c-myc	mouse	ab32	abcam
ß-tubulin	mouse	556321	BD Pharmingen
GAPDH	rabbit	E1C604	Enogene

secondary antibodies

**against:**	**host**	**Cat #**	**company**
mouse IgG	rabbit	HAF007	R & D
rabbit IgG	goat	31460	Thermo Scientific
goat IgG	rabbit	HAF017	R & D

siRNA

**description**	**Cat #**	**company**
FOXP1-HSS120361	10620312	invitrogen
FOXP1-HSS120362	10620312	invitrogen
FOXP1-HSS120363	10620312	invitrogen

cDNA Clones

**gene**	**Cat #**	**company**
Sema5b (Myc-DDK-tagged)	RC209657	Origene
FoxP1	TC103781	Origene
